# Magnetic resonance imaging as a clue to successful diagnosis of renal tuberculosis: a case report

**DOI:** 10.4076/1757-1626-2-8879

**Published:** 2009-08-12

**Authors:** Nobuaki Matsui, Tatsuo Morita

**Affiliations:** Department of Urology, Jichi Medical University Hospital3311-1 Yakushiji, Shimotsuke, Tochigi, 3290498Japan

## Abstract

Computed tomography is considered as the imaging modality of choice in the diagnosis of genitourinary tuberculosis, while magnetic resonance imaging may provide some informative features corresponding to the pathologic stage of the disease. We herein present a case report where magnetic resonance imaging showed the informative features, and a clue to further examinations in focusing on renal tuberculosis.

## Case presentation

**Figure 1.**
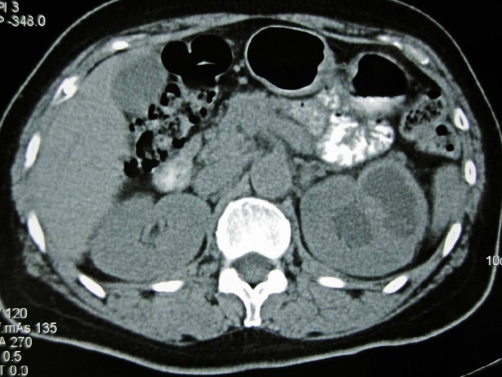
Plain CT image demonstrates a cystic mass lesion in the upper pole of the left kidney. No calcifications were detected.

**Figure 2.**
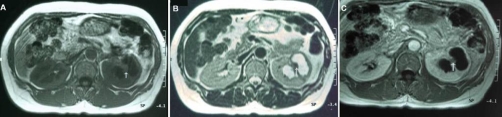
Transverse T1-weighted **(A)**, T2-weighted **(B)** and Gadolinium (Gd) -enhanced T1-weighted **(C)** images are presented. Left kidney has cystic mass lesion at upper pole, which is composed of thinned parenchyma and water signal component. The abnormal renal parenchyma is demonstrated with low signal intensity on both T1 and T2-weighted images. A small-sized parenchymal nodule (arrow) is also depicted isointense and hypointense on T1 and T2-weighted images, respectively, when compared to the normal renal parenchyma. The abnormal parenchyma is heterogeneously enhanced and the nodule is not enhancing after Gd administration.

A 53-year-old Japanese woman was referred to our institution for recurrent cystitis. Abdominal sonogram was initially performed, revealing the presence of a left upper pole cystic lesion in a hydronephrotic kidney and dilatation of the ipsilateral ureter. Subsequent CT examination, including both plain and contrast-enhanced images confirmed the presence of a cystic mass at upper pole of the left kidney, accompanied by thinning and enhancement of the renal parenchyma. The dimensions of the lesion were 40 × 25 mm. No renal calcifications were noted. Left ureteral dilatation was also seen, with thickening and enhancement of the ureteral wall (Figure 1). Based on the CT findings, differential diagnosis included chronic pyelonephritis, papillary necrosis, medullary sponge kidney, renal cell carcinoma, transitional cell carcinoma and renal tuberculosis were considered. We then performed MRI for further examination, which depicted the thinned parenchymal left kidney lesion, of low intensity on both T1 and T2-weighted images, when compared to the normal renal parenchyma, heterogeneously enhancing after gadolinium administration. In addition, there was a nodule in the parenchyma that was not enhanced by gadolinium administration with iso intensity on T1-weighted images and low intensity on T2-weighted images (Figure 2). MR imaging findings indicated inflammation and fibrosis with a granuloma formation, therefore strongly suggesting renal tuberculosis. Based on our findings, further examinations were scheduled in focusing on urinary tract tuberculosis. Subsequent cystoscopy showed reddish and edematous mucosa around left ureteral orifice and biopsied specimen revealed a granuloma. Urine culture and urine polymerase chain reaction (PCR) were positive for mycobacterium tuberculosis. Her tuberculin skin test was positive, showing 48 × 62 mm rubor with induration, whereas her chest radiograph showed no nodular lesion or cavity. Under the diagnosis of urinary tract tuberculosis, she received antitubercular medications with isoniazid, rifampicin, pyrazinamide and ethambutol for 9 months. Because of persistent pyuria and left kidney dysfunction, left nephroureterectomy was subsequently performed, although urine culture and PCR had become negative for mycobacterium tuberculosis. Pathological examinations of resected specimen revealed caseous necrotic granuloma and lymphocytes infiltration to the renal parenchyma and ureter, consisting with urinary tract tuberculosis. The patient is now well, without signs of disease recurrence five years after surgery.

## Discussion

The genitourinary tract is one of the most common sites of extrapulmonary tuberculosis, accounting for 15-20% of infections outside lungs and approximately 4-8% with pulmonary tuberculosis will develop significant genitourinary tuberculosis [1]. The diagnosis is made on basis of urine culture studies or histopathologic analysis and is supported by radiologic studies. The findings of the disease on plain abdominal radiography are calcifications. Renal calcifications are a one of the common manifestations of tuberculosis at conventional radiography, occurring in 24-44% [2]. Intravenous urography may show a variety of findings, including moth-eaten calyces, amputated infundibula, hydronephrosis or hydronephroureter due to ureteral strictures and non-function of a kidney [3,4]. CT is the most sensitive modality for renal calcifications which occur in over 50% of cases of genitourinary tuberculosis [2,5] and is thought to be a mainstay in the diagnostic images of renal tuberculosis, showing renal parenchymal cavity, mass and scaring, local parenchymal thinning, stricture of infundibula [4,6]. CT urography is a relatively new imaging examination which can provide comprehensive evaluation of both renal parenchyma and urothelium. Thus, sings of ureteral and bladder involvement by the disease may be nicely depicted [3]. The most useful radiologic feature of urinary tract tuberculosis is the multiplicity of abnormal findings [2], therefore the diagnosis of the disease can be suggested by a single examination (CT urography). Although MRI is reportedly of limited value in diagnostic images, it has an ability to depict the characteristic features corresponding to the subtle pathological changes and has some advantages including no radiation burden, detective capability of dilated collecting system, calyx and ureteral strictures independent of renal function [3,7]. In present case, these findings of local parenchymal thinning and dilated calices might be pathologically ascribed to characteristic tubercular changes, not only inflammation, fibrosis and destructive caseous necrosis in the renal parenchyma, but the stricture by fibrosis at ureter and/or calyx infundibula. Furthermore, the nodule in the renal parenchyma had isointensity on T1-weighted images and low intensity on T2-weighted images without gadolinium enhancement. This nodule seemed to correspond to a caseous necrotic granuloma found in the resected specimen. Thus MRI provided us a clue to further examinations in focusing on urinary tract tuberculosis leading to successful diagnosis. Although CT is indeed the most common modality in diagnostic images, the diagnosis cannot be made by a single examination and requires multi-modality methods [4]. Present case suggests that MRI could provide more informative features of renal tuberculosis, especially in case of no calcifications.

## Abbreviations

CT: computed tomography
MRI: magnetic resonance imaging
PCR: polymerase chain reaction
